# Serum levels of ferritin do not affect the prognosis of patients with hepatocellular carcinoma undergoing radiofrequency ablation

**DOI:** 10.1371/journal.pone.0200943

**Published:** 2018-07-25

**Authors:** Koji Uchino, Ryosuke Tateishi, Ryo Nakagomi, Naoto Fujiwara, Tatsuya Minami, Masaya Sato, Kenichiro Enooku, Hayato Nakagawa, Yoshinari Asaoka, Yuji Kondo, Junji Shibahara, Shuichiro Shiina, Kazuhiko Koike

**Affiliations:** 1 Department of Gastroenterology, Graduate School of Medicine, The University of Tokyo, Tokyo, Japan; 2 Department of Pathology, Kyorin University Schoole of Medicine, Tokyo, Japan; 3 Department of Gastroenterology, Juntendo University, Tokyo, Japan; Nihon University School of Medicine, JAPAN

## Abstract

**Background & aims:**

Hepatic iron accumulation can accelerate liver injury in patients with various chronic liver diseases and lead to hepatocarcinogenesis. We elucidated the impact of serum levels of ferritin on the prognosis of hepatocellular carcinoma (HCC) after radiofrequency ablation (RFA) in a large cohort.

**Methods:**

We retrospectively analyzed 578 treatment-naïve HCC patients who underwent RFA. We divided our cohort into four groups by the quartile points of serum ferritin level: G1 (≤55 ng/mL, n = 148), G2 (56–130 ng/mL, n = 142), G3 (131–243 ng/mL, n = 144) and G4 (≥244 ng/mL, n = 144). We analyzed the recurrence and survival of patients using the Kaplan–Meier method. We also evaluated pathological iron deposition among patients with a solitary tumor smaller than 2 cm.

**Results:**

The cumulative rates of overall recurrence and survival at 5 years were 81.6% and 66.3%, respectively. The serum levels of ferritin were correlated with pathological iron deposition. There were no significant differences in recurrence and survival rates according to serum levels of ferritin and pathological hepatic iron deposition.

**Conclusions:**

Serum levels of ferritin do not affect the prognosis of HCC patients undergoing RFA.

## Introduction

Hepatocellular carcinoma (HCC) is a common cause of cancer-related deaths worldwide.[1] HCC usually develops in a liver already suffering from chronic diseases such as chronic viral hepatitis, alcoholic liver disease, and nonalcoholic fatty liver disease (NAFLD).[2, 3]

Hepatic iron accumulation is often present in patients with various chronic liver diseases including hemochromatosis, chronic hepatitis C, and NAFLD, and is thought to contribute to liver injury in these patients.[4–6] Excess iron injures cells primarily by catalyzing the production of reactive oxygen species, which cause lipid peroxidation, oxidation of amino acids with consequent protein-protein cross-linking, protein fragmentation, and DNA damage.[7] Recent studies have suggested that these oxidative stresses play a key role in liver injury and hepatocarcinogenesis.[8–13] In addition, phlebotomy improves elevated serum transaminase levels and histological changes, and may reduce the risk of HCC development in patients with chronic hepatitis C.[14–16] Hepatic iron overload is evaluable using a biopsy specimen for either histological or direct measurement of hepatic iron concentration.[17] However, liver biopsy is an invasive procedure with the possibility of life-threatening complications.[18]

Serum ferritin is widely used to assess iron homeostasis.[7] We previously reported that extremely high or low serum levels of ferritin are an independent risk factor for HCC development in male patients with chronic hepatitis C.[19] Moreover, they are a predictor of mortality in patients with end-stage liver disease, before and after liver transplantation.[20–22] Recently, serum ferritin was found to be one of the predictors of disease progression and long-term mortality in patients with NAFLD[23–25]. However, few studies have assessed its impact on the prognosis of HCC patients after curative treatment.

Radiofrequency ablation (RFA) along with resection is recommended as a standard curative treatment option for early-stage HCC because of its high local controllability.[26–29] We elucidated the impact of serum levels of ferritin and pathological hepatic iron accumulation on the prognosis of HCC patients undergoing RFA in a large cohort.

## Materials and methods

### Patients

From January 2006 to December 2010, 622 treatment-naïve HCC patients underwent RFA as initial treatment at the Department of Gastroenterology, the University of Tokyo Hospital. We enrolled 611 of these patients in the current study after excluding 11 patients in whom RFA was performed with non-curative intent to reduce tumor burden. Of those 611 patients, we enrolled 578 (94.6%) in this study in whom serum levels of ferritin were measured on admission before RFA; these patients were subjected to a retrospective analysis. The inclusion criteria for RFA were as follows: total bilirubin level of less than 3 mg/dL, platelet count of no less than 50 ⨉ 10^3^/mm^3^, and prothrombin activity level of no less than 50%. Patients with macroscopic vascular invasion, refractory ascites, or extrahepatic metastasis were excluded. In general, we performed RFA on patients with three or fewer lesions of 3 cm or less in diameter. We also performed ablation on patients beyond these criteria if it was predicted to be clinically effective.[30, 31] We enrolled patients who underwent transcatheter arterial chemoembolization prior to RFA when the treatments were performed sequentially. If possible, a background liver biopsy was performed simultaneously with RFA. This study was conducted according to the ethical guidelines for epidemiologic research developed by the Ministry of Education, Culture, Sports, Science and Technology and the Ministry of Health, Labour and Welfare, Japan. The study design was approved by the University of Tokyo Medical Research Center Ethics Committee (Registration No. 2058).

### Diagnosis of HCC

HCC was diagnosed by dynamic computed tomography (CT), and hyperattenuation in the arterial phase with washout in the late phase was considered the definite sign of HCC.[32, 33] When the diagnosis of HCC was not definite, ultrasound-guided tumor biopsy was performed, and pathological diagnosis was made based on the Edmondson-Steiner criteria.[34]

### Treatment and evaluation

RFA was performed on an in-patient basis. The details of the RFA technique are described elsewhere.[28] Briefly, a 17-gauge, cooled-tip electrode (Covidien, Mansfield, MA, USA) was inserted into the lesion under real-time ultrasound guidance. We started ablation at 60 W for the 3 cm exposed tip and 40 W for the 2 cm exposed tip. The power was increased to 140 W at a rate of 20 W/min. When a rapid increase in impedance was observed during thermal ablation, we minimized the output for 15 s and restarted the emission at a lower output. The duration of a single ablation was 12 min for the 3 cm electrode and 6 min for the 2 cm electrode. After one or two sessions of RFA, dynamic CT was performed to evaluate the treatment efficacy. A lesion was judged to be completely ablated when the non-enhanced area shown in the late phase of CT post-ablation covered the entire lesion shown in both the early and late phases of CT pre-ablation with a safety margin in the surrounding liver parenchyma. We confirmed complete ablation in all of the slices on which the target nodule was visualized. Patients received additional sessions until complete ablation was confirmed in each nodule.

### Follow-up

The follow-up regimen consisted of blood tests and monitoring of tumor markers in an outpatient setting. Ultrasonography and dynamic CT were also performed every 4 months. Tumor recurrence was defined as a newly developed lesion on a dynamic CT that showed hyperattenuation in the arterial phase with washout in the late phase. Local tumor progression was defined as the appearance of viable cancer tissue touching the initially treated tumor and distant recurrence as the emergence of one or several tumors separate from the primary site.[35] RFA was performed for recurrent tumors if the patient still met the indication criteria. If multiple recurrences were not treatable with RFA, transarterial chemoembolization was generally performed.

### Pathological evaluation of hepatic iron accumulation

**Fig 1:**
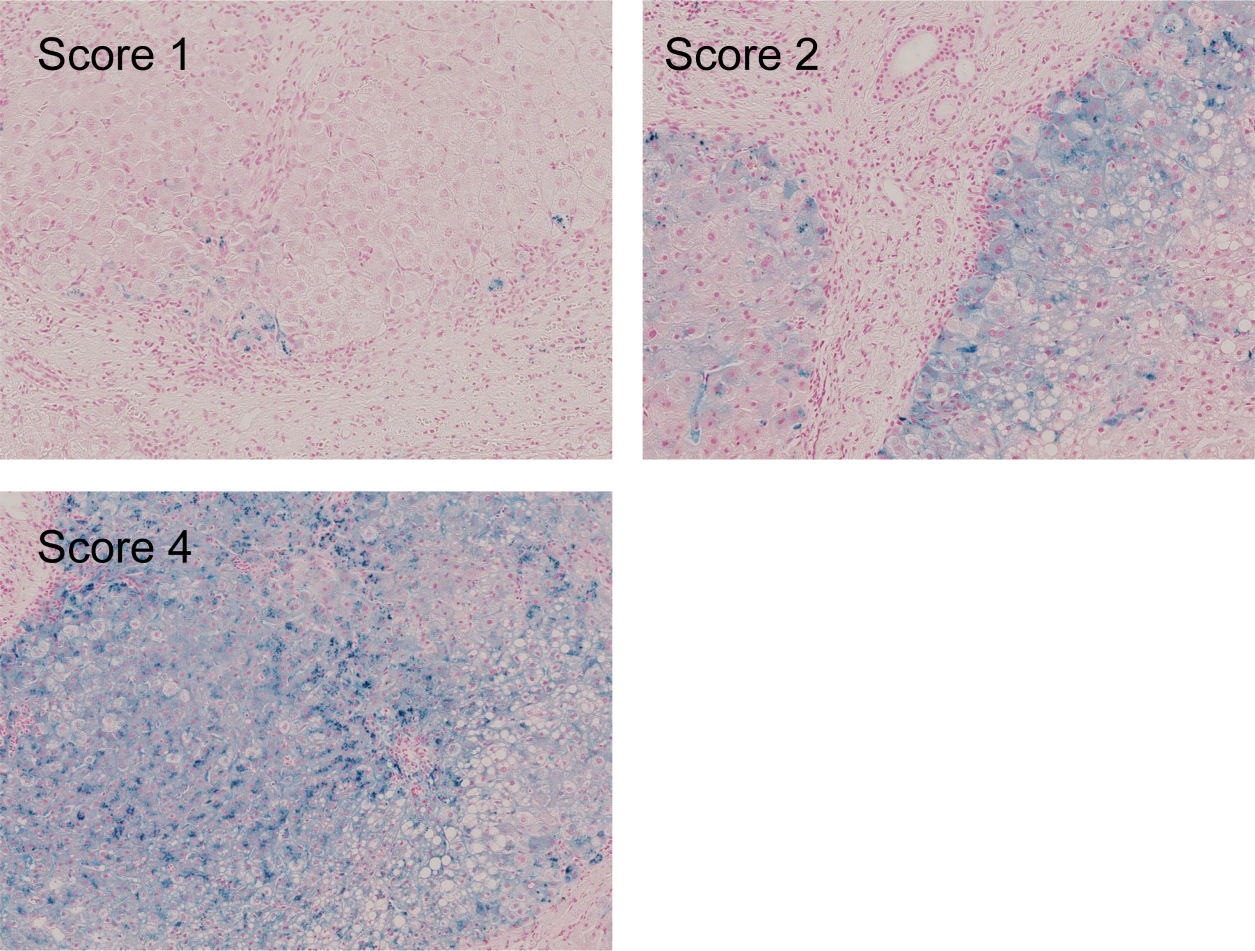
Scoring of pathological hepatic iron accumulation. (A) a score of 1 was assigned to biopsies with hemosiderin in rare periportal hepatocytes or at the periphery of regenerative parenchymal nodules; (B) a score of 2 was assigned to biopsies with hemosiderin in numerous periportal hepatocytes in zone 1 of the hepatic acini or extensively around the periphery of regenerative parenchymal nodules; (C) a score of 4 was assigned to biopsies with hemosiderin throughout all of the acinar zones or throughout regenerative parenchymal nodules.

Background liver biopsy was performed on the same day of RFA whenever possible. Biopsy specimens of 22 mm in lenght were obtained using a 16-gauge biopsy needle (Bard Monopty, Bard Peripheral Vascular, Inc., Tempe, AZ, USA). Iron accumulation in the non-cancerous liver parenchyma was evaluated pathologically using the liver biopsy specimen. Prussian blue staining was applied to evaluate hepatic iron deposition. Of patients with a solitary tumor smaller than 2 cm, hepatic iron accumulation was evaluated in detail by a pathologist with 15 years of experience in liver pathology using the semi-quantitative method described by Scheuer and Lefkowitch; for example, a score of 1 was assigned to biopsies with hemosiderin in rare periportal hepatocytes or at the periphery of regenerative parenchymal nodules, a score of 2 was assigned to biopsies with hemosiderin in numerous periportal hepatocytes in zone 1 of the hepatic acini or extensively around the periphery of regenerative parenchymal nodules, a score of 3 was given to biopsies with hemosiderin extensively in zones 1 and 2 of the hepatic acini or more extensively in regenerative parenchymal nodules, and a score of 4 was assigned to biopsies with hemosiderin throughout all of the acinar zones or throughout regenerative parenchymal nodules.[5, 36] The representative pictures of each socre were shown in Fig 1. The patients’ clinicopathological information was concealed from the pathologist. We hypothesized that hepatic iron accumulation influences the recurrence caused by multicentric carcinogenesis (i.e., *de novo*, not intrahepatic metastasis). Thus, we evaluated hepatic iron accumulation semi-quantitatively only in patients with a solitary small HCC because they were less likely to have intrahepatic metastasis. Along with evaluation of hepatic iron accumulation, we also evaluated stage of liver fibrosis and grade of necroinflammatory activity with METAVIR system.[37, 38]

### Stratification according to serum ferritin level

We divided our cohort into the following four groups by the quartile points of serum ferritin level: G1 (≤55 ng/mL, n = 148), G2 (56–130 ng/mL, n = 142), G3 (131–243 ng/mL, n = 144), and G4 (≥244 ng/mL, n = 144).

### Statistical analysis

The categorical variables were compared by the Fisher’s exact test, whereas continuous variables were compared by one-way analysis of variance (parametric) or the Kruskal–Wallis test (non-parametric). The cumulative survival rate and incidence of HCC recurrence were estimated using the Kaplan–Meier method. Follow-up ended on December 31, 2015. For prognostic factors, we used the following variables together with serum ferritin level in univariate and multivariate Cox proportional hazard regression analyses: age, sex, hepatitis B surface antigen positivity, hepatitis C antibody positivity, alcohol consumption, body mass index (BMI), aspartate aminotransferase (AST) level, hemoglobin, platelet count, Child-Pugh classification, tumor size, tumor number, alpha-fetoprotein (AFP) level, *lens-culinaris* agglutinin-reactive fraction of AFP (AFP-L3) level, and des-γ-carboxyl-prothrombin (DCP) level. Stepwise variable selection with the Akaike information criterion was used to identify the best model in multivariate analyses. *P* values of < 0.05 in a two-tailed test were considered statistically significant. All of the statistical analyses were performed using R 3.4.3 (http://www.R-project.org).

## Results

### Patient profiles

The enrolled HCC patient cohort in this study consisted of 369 males and 209 females with a median age of 70.6 years. The median tumor size was 2.2 cm. A total of 369 patients (62.8%) had a solitary HCC lesion. The median serum ferritin level was 129.5 (25–75th percentiles: 55.0–243.0) ng/mL. The age, sex, BMI, total bilirubin, AST, alanine aminotransferase (ALT), hemoglobin, platelet count, tumor number, AFP level, and AFP-L3 level differed among the four groups (Table 1).

**Table 1:** Baseline characteristics of the patients (n = 578).

Variables	Overall (n = 578)	G1 (n = 148)	G2 (n = 142)	G3 (n = 144)	G4 (n = 144)	P value
Age (years) *	70.6 (63.4–76.0)	71.6 (63.0–76.2)	72.5 (65.2–76.7)	70.5(64.6–76.2)	68.4 (61.3–73.4)	0.01^§^
Male sex, n (%)	369 (63.8)	87 (58.8)	77 (54.2)	96(66.7)	109 (75.7)	0.0008^‡^
Viral infection, n(%)						
HBsAg positive only	79 (13.7)	15 (10.1)	24 (16.9)	25 (17.4)	15 (10.4)	0.12^‡^
Anti HCVAb positive only	406 (70.2)	102 (68.9)	104 (73.2)	96 (66.7)	104 (72.2)	0.60^‡^
Both positive	2 (0.3)	2 (1.4)	0 (0)	0 (0)	0 (0)	0.25^‡^
Both negative	91 (15.7)	29 (19.6)	14 (9.9)	23 (16.0)	25 (17.4)	0.11^‡^
Alcohol consumption >80 g/day, n (%)	81 (14.0)	18 (12.2)	16 (11.3)	23 (16.0)	24 (16.7)	0.46^‡^
Body mass index (kg/m^2^)*	23.2 (21.2–25.3)	23.1 (20.8–25.1)	22.5 (20.1–24.4)	23.5 (21.2–25.6)	23.8 (21.6–26.2)	0.01^§^
Serum albumin (g/dL)*	3.7 (3.4–4.0)	3.7 (3.4–4.0)	3.7 (3.4–4.1)	3.8 (3.4–4.1)	3.6 (3.3–3.9)	0.12^§^
Total bilirubin (mg/dL)*	0.8 (0.6–1.1)	0.8 (0.6–1.1)	0.8 (0.6–1.1)	0.9 (0.7–1.2)	0.9 (0.7–1.3)	0.0005^§^
AST (IU/L)*	50 (36–71)	44 (33–60)	47 (31–65)	50 (37–68)	65 (47–89)	<0.0001^§^
ALT (IU/L)*	44 (28–65)	32 (24–47)	39 (27–55)	47 (32–63)	69 (44–99)	<0.0001^§^
Prothrombin activity (%)*	90 (76–100)	91 (77–100)	92 (79–100)	88 (76–100)	90 (74–100)	0.21^§^
Hemoglobin (g/dL)*	13.0 (11.9–14.0)	12.1 (10.9–13.2)	12.9 (11.9–13.8)	13.4 (12.5–14.2)	13.5 (12.4–14.4)	<0.0001^§^
Platelet count (× 10^9^/L)*	104 (76–142)	110 (77–158)	109 (79–146)	112 (86–158)	94 (68–121)	0.0005^§^
Child-Pugh classification, n(%)						0.15^‡^
Class A	465 (80.4)	112 (75.7)	121 (85.2)	116 (80.6)	116 (80.6)	
Class B	108 (18.7)	32 (21.6)	21 (14.8)	27 (18.8)	28 (19.4)	
Class C	5 (0.9)	4 (2.7)	0 (0)	1 (0.7)	0 (0)	
Tumor size (cm)*	2.2 (1.8–2.8)	2.1 (1.7–2.8)	2.3 (1.8–2.6)	2.2 (1.7–2.9)	2.3 (1.9–2.9)	0.42^§^
Tumor number						0.048^‡^
Solitary, n(%)	363 (62.8)	102 (68.9)	92 (64.8)	92 (63.9)	77 (53.5)	
Two or more, n(%)	215 (37.2)	46 (31.1)	50 (35.2)	52 (36.1)	67 (46.5)	
AFP >100 ng/mL, n (%)	97 (16.8)	15 (10.1)	36 (25.4)	20 (13.9)	26 (18.1)	0.005^‡^
DCP >100 mAU/mL, n (%)†	99 (17.1)	19 (12.8)	28 (19.7)	25 (17.4)	27 (18.8)	0.40^‡^
AFP-L3 >15%, n (%)	69 (11.9)	25 (16.9)	23 (16.2)	12 (8.3)	9 (6.2)	0.006^‡^

* Expressed as medians (25–75th percentiles).

† missing in 11 patients.

‡ Fisher’s exact test.

§ Kruskal-Wallis test.

HBsAg, hepatitis B surface antigen; anti-HCVAb, anti-hepatitis C virus antibody; AST, aspartate aminotransferase; ALT, alanine aminotransferase; AFP, alpha-fetoprotein; DCP, des-γ-carboxyl-prothrombin; AFP-L3, *lens-culinaris* agglutinin-reactive fraction of AF

### Relationship between serum levels of ferritin and other clinical factors

**Fig 2:**
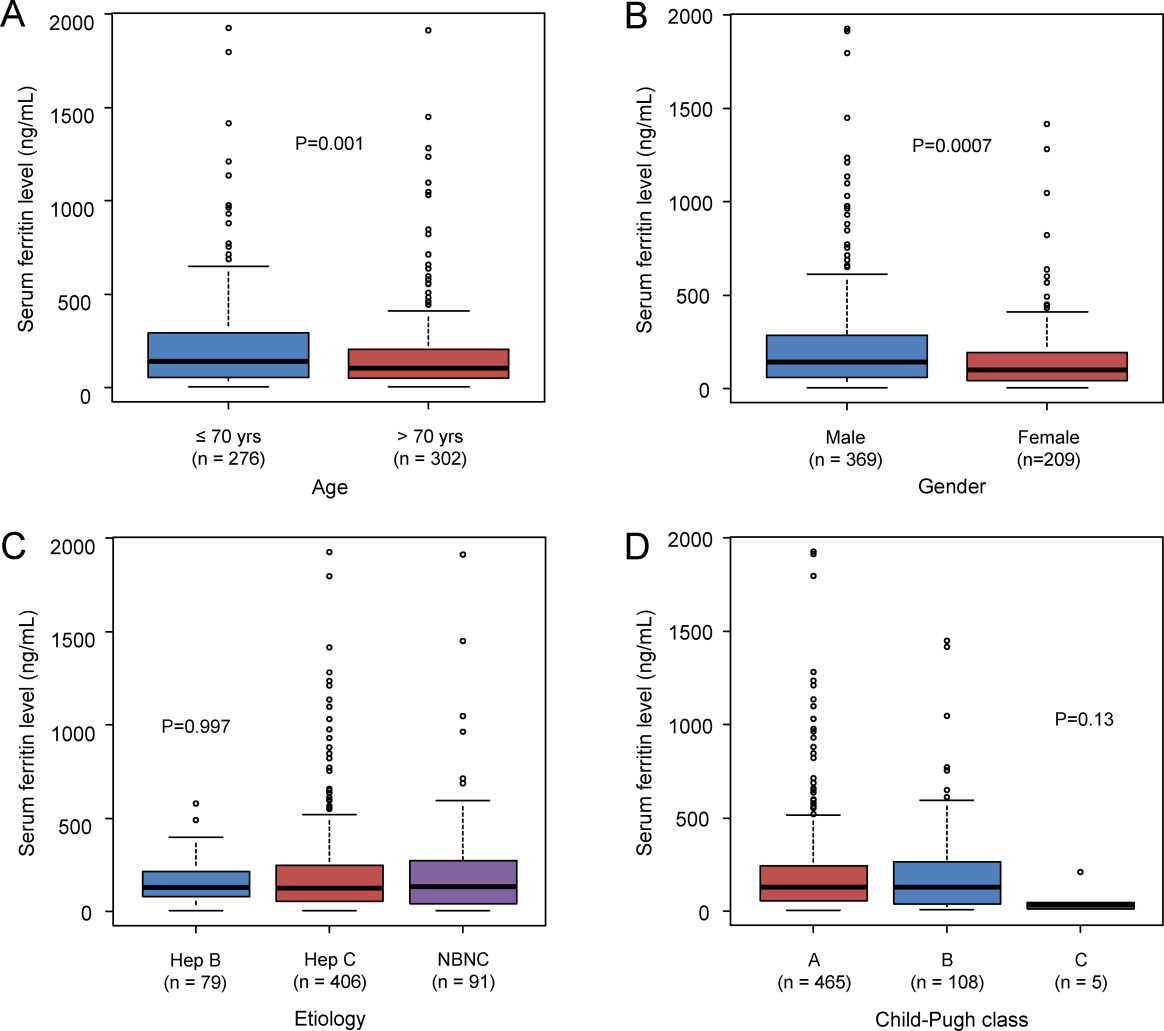
Relationship between serum levels of ferritin and other clinical factors. (A) Age, (B) Sex, (C) Etiology: hepatitis B related, hepatitis C related or non-B non-C, and (D) Child-Pugh classification.

The median serum level of ferritin was 143.5 ng/mL in patients aged 70 or younger and 106.5 ng/mL in patients over 70 years of age. Serum levels of ferritin in patients over 70 years of age were significantly lower than those of patients aged 70 or younger (P = 0.001) (Fig 2A). Serum levels of ferritin were significantly lower in female than in male (median: 142.0 ng/mL in male and 103.0 ng/mL in female, P = 0.0007) (Fig 2B). The median serum level of ferritin was 131.0 ng/mL in hepatitis B patients, 128.0 ng/mL in hepatitis C patients and 134.0 ng/mL in non-B non-C patients, respectively. According to the etiology, there was no statistical difference in serum levels of ferritin (P = 0.997) (Fig 2C). The median serum level of ferritin was 130.0 ng/mL in Child-Pugh class A patients, 131.0 ng/mL in class B patients, 48 ng/mL in class C patients. Although threre was no significant difference, the serum levels of ferritin were lower in Child-Pugh class C patients than in class A or B patients (P = 0.13) (Fig 2D).

### Serum levels of ferritin according to the hepatic iron accumulation score

**Fig 3:**
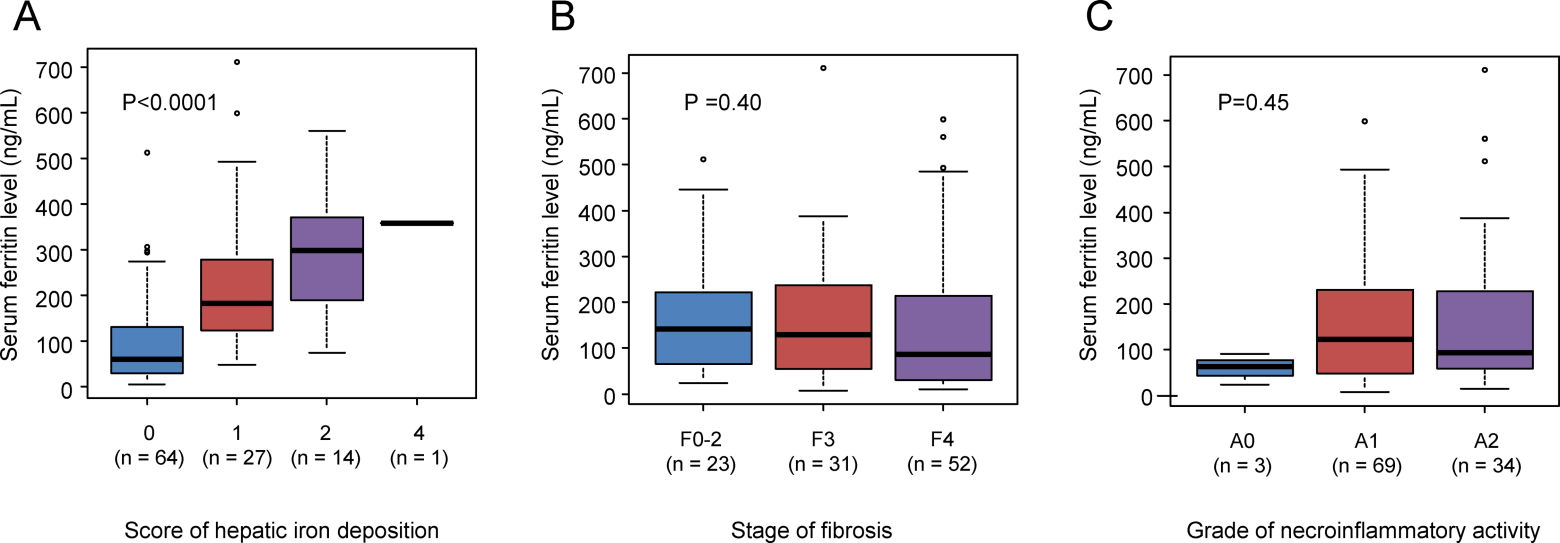
Serum levels of ferritin according to pathological findings. (A) According to pathological hepatic iron accumulation socore, (B) stage of fibrosis, and (C) grade of necroinflammatory activity in METAVIR system.

In our cohort, 136 patients had solitary HCC less than 2 cm (very early stage of BCLC staging system). Of those 136 patients, hepatic iron accumulation was evaluable pathologically in 106 patients. In total, 64 patients were assigned a score of 0, 27 patients were assigned a score of 1, 14 patients had a score of 2, and 1 patient was assigned a score of 4. No patient was assigned a score of 3. The median serum levels of ferritin were 59.0, 184.0, 272.5 and 359.0 ng/mL in patients with scores of 0, 1, 2, and 4, respectively (Fig 3A), with statistically significant differences (P< 0.0001). The serum levels of ferritin in patients with a score of 1 or 2 were significantly higher than those in patients with a score of 0 (P < 0.0001 and P < 0.0001, respectively). The serum levels of ferritin were not significantly different according to the stage of liver fibrosis and grade of necroinflammatory activity in METAVIR system (Fig 3B and 3C).

### HCC recurrence

**Fig 4:**
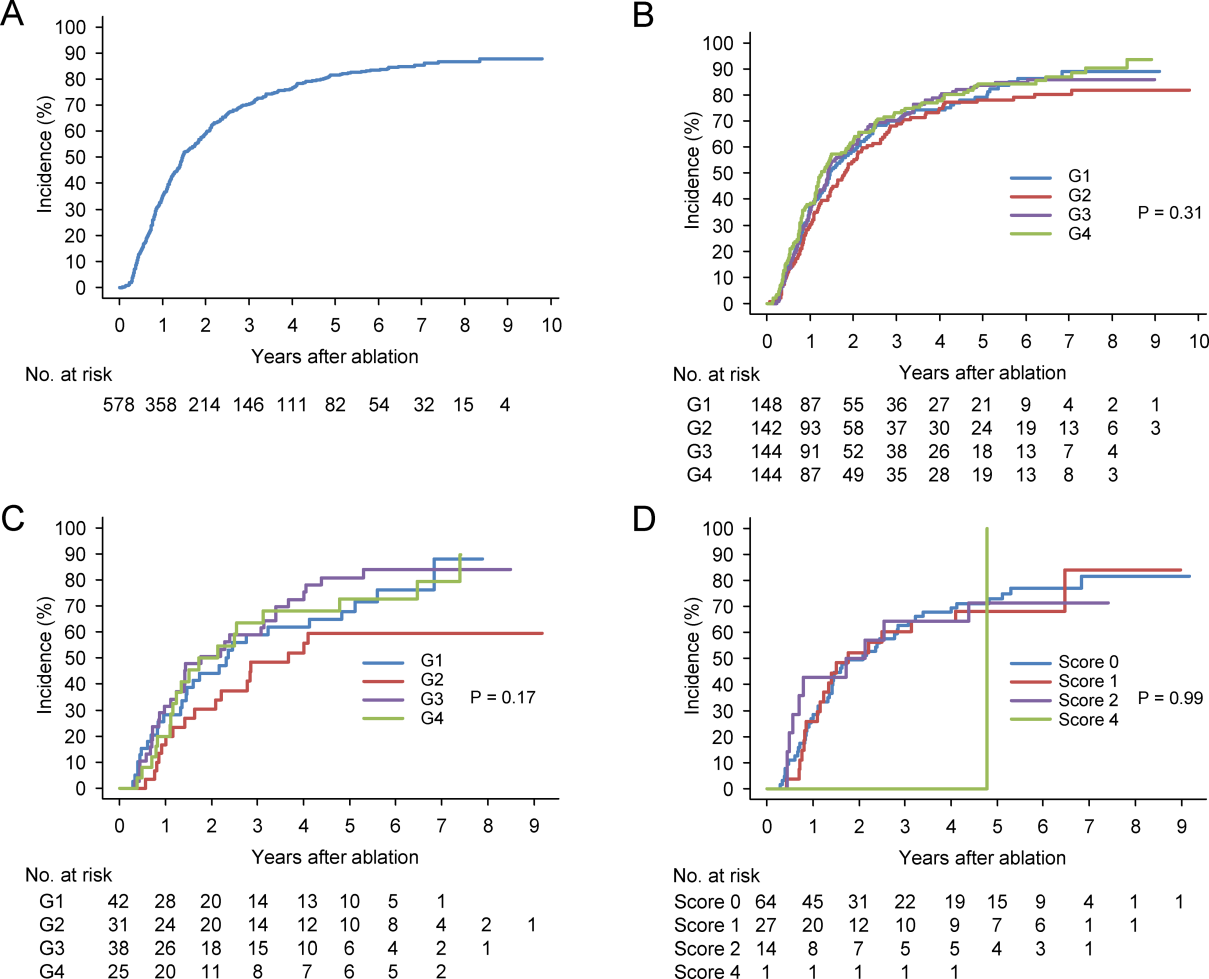
Cumulative recurrence of hepatocellular carcinoma (HCC) patients after radiofrequency ablation. (A) Entire cohort, (B) according to serum ferritin level, (C) according to serum ferritin level among patients with a solitary HCC lesion of less than 2 cm, and (D) according to pathological hepatic iron accumulation score among patients with a solitary HCC lesion of less than 2 cm.

During the follow-up period, tumor recurrence was identified in 455 cases. The cumulative rates of overall recurrence at 1, 3, and 5 years were 35.3%, 70.7%, and 81.6%, respectively (Fig 4A). The cumulative rates of overall recurrence at 5 years in G1, G2, G3, and G4 were 79.2%, 78.3%, 83.9%, and 84.4%, respectively. There were no significant differences in the recurrence rate among the four groups (Fig 4B, P = 0.31). When we only analyzed cases in the very early stage of BCLC staging system, there were no significant differences in recurrence rates according to serum ferritin level (Fig 4C, P = 0.17) or hepatic iron accumulation (Fig 4D, P = 0.99). Univariate analyses demonstrated that sex, hepatitis B surface antigen, hepatitis C antibody, AST level, platelet count, Child-Pugh classification, tumor number, and AFP-L3 level were correlated with recurrence (Table 2). A multivariate analysis showed that age, sex, AST, hemoglobin, Child-Pugh class, tumor number, and AFP-L3 level, but not serum ferritin level, were independent predictors of recurrence.

**Table 2:** Univariate and multivariate analyses of variables related to recurrence.

	Univariate		Multivariate	
Variables	Hazard ratio (95%CI)	P value	Hazard ratio (95%CI)	P value
Age (per 1 year)	1.01 (1.00–1.02)	0.11	1.01 (1.00–1.02)	0.04
Male sex	1.23 (1.01–1.50)	0.04	1.51 (1.22–1.89)	0.0002
HBsAg positive	0.70 (0.53–0.92)	0.01		
Anti HCVAb positive	1.32 (1.07–1.62)	0.01		
Alcohol consumption				
≤80 g/day	1			
>80 g/day	1.19 (0.91–1.55)	0.22		
Body mass index (per 1 kg/m^2^)	1.01 (0.98–1.04)	0.55		
AST				
≤50 IU/L	1		1	
>50 IU/L	1.64 (1.36–1.97)	<0.0001	1.63 (1.34–1.98)	<0.0001
Hemoglobin (per 1 g/dL)	0.94 (0.89–1.00)	0.06	0.93 (0.87–0.99)	0.03
Platelet count				
≤10^11^ /L	1		1	
>10^11^ /L	0.75 (0.63–0.91)	0.003	0.85 (0.70–1.04)	0.12
Child-Pugh classification				
Class A	1			
Class B or C	1.62 (1.28–2.05)	<0.0001	1.38 (1.07–1.78)	0.01
Tumor size (per 1 mm)	1.01 (1.00–1.02)	0.09		
Tumor number				
Solitary	1		1	
Two or more	1.74 (1.44–2.10)	<0.0001	1.74 (1.43–2.11)	<0.0001
AFP				
≤100 ng/mL	1			
>100 ng/mL	1.26 (0.99–1.60)	0.06		
DCP				
≤100 mAU/mL	1		1	
>100 mAU/mL	1.14 (0.89–1.45)	0.31	1.23 (0.96–1.59)	0.10
AFP-L3				
≤15%	1		1	
>15%	1.44 (1.09–1.89)	0.009	1.60 (1.21–2.13)	0.001
Serum ferritin				
G1 (≤55 ng/mL)	1			
G2 (56–130 ng/mL)	0.86 (0.66–1.12)	0.27		
G3 (131–243 ng/mL)	1.03 (0.80–1.34)	0.82		
G4 (≥244 ng/mL)	1.10 (0.85–1.43)	0.45		

CI, confidence interval; HBsAg, hepatitis B surface antigen; anti-HCVAb, anti-hepatitis C virus antibody; AST, aspartate aminotransferase; AFP, alpha-fetoprotein; DCP, des-γ-carboxyl-prothrombin; AFP-L3, *lens culinaris* agglutinin-reactive fraction of AFP.

### Survival

**Fig 5:**
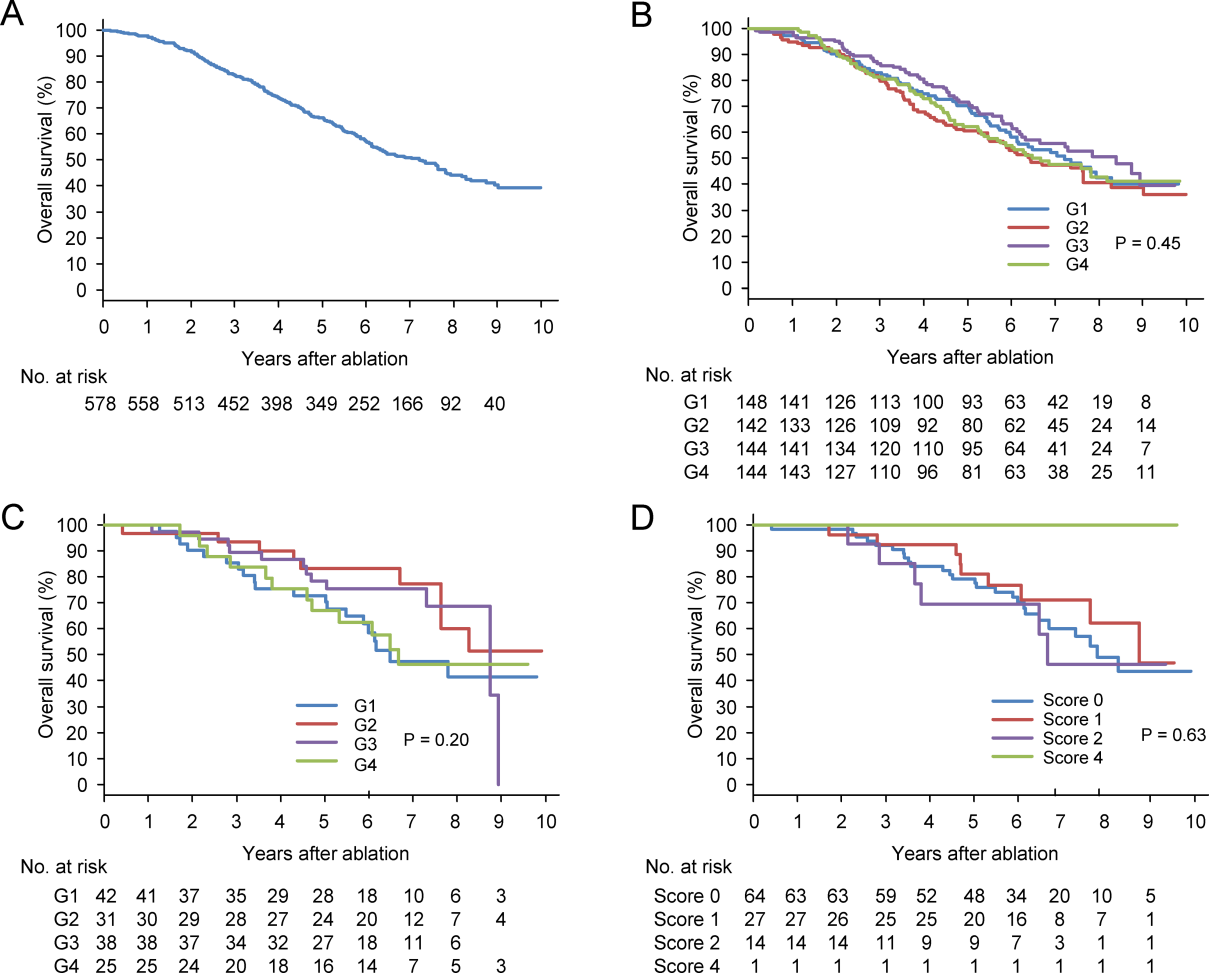
Cumulative overall survival of hepatocellular carcinoma (HCC) patients after radiofrequency ablation. (A) Entire cohort, (B) according to serum ferritin level, (C) according to serum ferritin level among patients with a solitary HCC lesion of less than 2 cm, and (D) according to pathological hepatic iron accumulation score among patients with a solitary HCC lesion of less than 2 cm.

During the follow-up period, 278 patients died. The cumulative rates of overall survival at 1, 3, 5, 7, 9 years were 97.7%, 82.7%, 66.3%, 50.8%, and 40.4%, respectively (Fig 5A). The cumulative rates of overall survival at 5 years in G1, G2, G3, and G4 were 70.4%, 60.5%, 71.8%, and 62.3%, respectively. There were no significant differences in survival rates among the four groups according to serum ferritin level (Fig 5B; P = 0.45). When we only analyzed cases at a very early stage of BCLC staging system, again there were no significant differences in survival rate according to serum ferritin level (Fig 5C, P = 0.20) or hepatic iron accumulation (Fig 5D, P = 0.63). Univariate analyses demonstrated that age, hepatitis B surface antigen, hepatitis C antibody, alcohol consumption, AST level, hemoglobin level, platelet count, Child-Pugh classification, tumor size, tumor number, and DCP level were correlated with survival (Table 3). A multivariate analysis showed that age, hepatitis B surface antigen positivity, alcohol consumption, AST, platelet count, Child-Pugh class, tumor number, DCP level, and AFP-L3 level, but not serum ferritin level, were independent predictors of survival.

**Table 3:** Univariate and multivariate analyses of variables related to survival.

	Univariate		Multivariate	
Variables	Hazard ratio (95%CI)	P value	Hazard ratio (95%CI)	P value
Age (per 1 year)	1.04 (1.02–1.05)	<0.0001	1.04 (1.02–1.06)	<0.0001
Male sex	1.06 (0.83–1.36)	0.63	1.27 (0.97–1.67)	0.08
HBsAg positive	0.29 (0.18–0.47)	<0.0001	0.45 (0.27–0.75)	0.002
Anti HCVAb positive	1.48 (1.13–1.94)	0.004		
Alcohol consumption				
≤80 g/day	1		1	
>80 g/day	1.59 (1.17–2.15)	0.003	1.42 (1.03–1.97)	0.03
Body mass index (per 1 kg/m^2^)	1.01 (0.98–1.05)	0.56		
AST (IU/L)				
≤50 IU/L	1		1	
>50 IU/L	1.56 (1.23–1.98)	0.0003	1.35 (1.06–1.73)	0.02
Hemoglobin (per 1 g/dL)	0.89 (0.83–0.95)	0.001		
Platelet count				
≤10^11^ /L	1		1	
>10^11^ /L	0.58 (0.46–0.73)	<0.0001	0.64 (0.49–0.83)	0.0008
Child-Pugh classification				
Class A	1		1	
Class B or C	2.34 (1.80–3.05)	<0.0001	2.13 (1.60–2.84)	<0.0001
Tumor size (per 1 cm)	1.01 (1.00–1.03)	0.03	1.01 (1.00–1.02)	0.14
Tumor number				
Solitary	1		1	
Two or more	1.36 (1.07–1.73)	0.01	1.33 (1.04–1.70)	0.02
AFP				
≤100 ng/mL	1			
>100 ng/mL	1.27 (0.94–1.71)	0.12		
DCP				
≤100 mAU/mL	1		1	
>100 mAU/mL	1.54 (1.15–2.05)	0.003	1.41 (1.04–1.92)	0.03
AFP-L3				
≤15%	1		1	
>15%	1.33 (0.95–1.87)	0.10	1.43 (1.01–2.03)	0.046
Serum ferritin				
G1 (≤55 ng/mL)	1			
G2 (56–130 ng/mL)	1.14 (0.83–1.58)	0.42		
G3 (131–243 ng/mL)	0.87 (0.62–1.23)	0.43		
G4 (≥244 ng/mL)	1.06 (0.76–1.48)	0.72		

CI, confidence interval; HBsAg, hepatitis B surface antigen; anti-HCVAb, anti-hepatitis C virus antibody; AST, aspartate aminotransferase; AFP, alpha-fetoprotein; DCP, des-γ-carboxyl-prothrombin; AFP-L3, *lens culinaris* agglutinin-reactive fraction of AFP.

## Discussion

The results of this study showed that serum level of ferritin reflect hepatic iron accumulation, but is not a prognostic factor for HCC patients after RFA. It has been already known that there were two different types of HCC recurrence, i.e. intrahepatic metastasis and de novo carcinogenesis.[39] As we have already described, hepatic iron accumulation is thought to contribute to liver injury and thus potentially hepatocarcinogenesis. Therefore, we hypothesized that high serum ferritin level and pathological hepatic iron accumulation are associated with HCC recurrence in the form of de novo carcinogenesis. In order to demonstrate the theory, we analyzed only in patients with a solitary small HCC who had lower risk of intrahepatic recurrence. However, the results were negative. Similar to previous reports in patients with chronic liver disease,[40, 41] serum ferritin level and pathological hepatic iron deposition score had significant positive correlations in our cohort. This confirms the validity of our evaluation of pathological hepatic iron deposition.

In our previous study, an extremely high or low serum ferritin level is an independent risk factor for HCC development in male patients with chronic hepatitis C.[19] However, in the current study, serum ferritin level was not a predictor of recurrence or survival among patients with HCC undergoing RFA. This result was unchanged when only male patients with HCV infection were analyzed. We assumed that serum ferritin level does not affect the prognosis of HCC patients undergoing RFA because of the greater impact of known prognostic factors, such as tumor factors and liver function.

By contrast, Facciorusso *et al*. reported that serum ferritin level is a risk factor for survival and recurrence after RFA in HCC patients.[42] The authors divided their cohort into two groups using a cutoff serum ferritin level of 244 ng/mL. The survival and recurrence rates of the patients with a serum ferritin level of > 244 ng/mL were significantly worse than those with a serum ferritin level of ≤ 244 ng/mL. Although we also used cutoff point of 244 ng/mL, there were no significant differences between the two groups (data not shown). The baseline characteristics of the patients such as age, sex, etiology, BMI, liver function and tumor stage were similar. The overall survival and recurrence rates were also similar. The main differences were as follows. First, our study involved a larger number of patients than did the previous work (578 vs. 103). Second, the median serum ferritin level was slightly lower in our cohort (129.5 vs. 188.5 ng/mL). Third, the vast majority of our patients were Japanese, while the previous study was conducted in Italy. These differences may create the discrepancy of the results between the two studies.

This study had several limitations. First, because this was retrospective study, the serum ferritin level in all HCC patients undergoing RFA during the study period could not be evaluated. However, we measured the serum ferritin levels of more than 90% of those patients. Thus, any selection bias was limited. Second, almost all of the patients in our cohort were Japanese, and the distribution of serum ferritin level differs among ethnicities.[43, 44] Third, data of other chemical markers related to iron metabolism, such as serum iron concentration and transferrin saturation, were missing. Forth, the majority of the patients in our cohort were HCV related HCC. Finally, we did not evaluate HFE mutation to rule out hereditary hemochromatosis; this was not essential because hereditary hemochromatosis is very rare in Japan (i.e., the prevalence of the C282Y mutation is 0.000039% among Asians).[43, 45]

In conclusion, serum levels of ferritin did not affect the prognosis of patients with HCC who underwent RFA.

## Supporting information

S1 File This is the dataset of this study. (XLSX)

## Abbreviations

AFP: alpha-fetoprotein
AFP-L3: *lens-culinaris* agglutinin-reactive fraction of AFP
AIC: Akaike information criterion
ALT: alanine aminotransferase
AST: aspartate aminotransferase
BCLC: Barcelona-Clinic-Liver-Cancer
BMI: body mass index
CT: computed tomography
DCP: des-γ-carboxyl-prothrombin
HCC: hepatocellular carcinoma
NAFLD: nonalcoholic fatty liver disease
RFA: radiofrequency ablation
